# Sonochemical Combined Synthesis of Nickel Ferrite and Cobalt Ferrite Magnetic Nanoparticles and Their Application in Glycan Analysis

**DOI:** 10.3390/ijms23095081

**Published:** 2022-05-03

**Authors:** Agnes Maria Ilosvai, Dalma Dojcsak, Csaba Váradi, Miklós Nagy, Ferenc Kristály, Béla Fiser, Béla Viskolcz, László Vanyorek

**Affiliations:** 1 Institute of Chemistry, University of Miskolc, Miskolc-Egyetemváros, 3515 Miskolc, Hungary; agnes.ilosvai.maria@uni-miskolc.hu (A.M.I.); kemfiser@uni-miskolc.hu (B.F.); bela.viskolcz@uni-miskolc.hu (B.V.); 2 Advanced Materials and Intelligent Technologies Higher Education and Industrial Cooperation Centre, University of Miskolc, 3515 Miskolc, Hungary; dalma.dojcsak@uni-miskolc.hu (D.D.); kemcsv@uni-miskolc.hu (C.V.); 3 Institute of Mineralogy and Geology, University of Miskolc, Miskolc-Egyetemváros, 3515 Miskolc, Hungary; askkf@uni-miskolc.hu

**Keywords:** magnetic, nanoparticles, ferrite, glycans, human serum

## Abstract

The combination of the sonochemical activation of Ni(NO_3_)_2_ and Co(NO_3_)_2_ in the presence of Fe(NO_3_)_3_ and polyethylene glycol and consecutive heat treatment of the formed metal hydroxides offers a cheap and efficient method for the preparation of nickel ferrite and cobalt ferrite magnetic nanoparticles, which can be successfully applied in the selective capture of fluorescently derivatized N-glycans from human serum. XRD measurement revealed that, besides the ferrite phase, nickel and cobalt oxides also form during heat treatment. The amount of simple metal oxides can be well controlled by the temperature of the heat treatment, since increasing temperature yielded higher spinel content. For both nickel and cobalt, the best heat treatment temperature was found to be 673 K, where the samples contained 84.1% nickel ferrite, and in the case of cobalt, almost pure (99.6%) cobalt ferrite could be prepared. FT-IR and zeta potential measurements indicated the presence of surface OH groups, which aided in the dispersion of the particles in water and, in addition, can promote the adsorption of polar compounds. The practical applicability of the magnetic nanopowders was demonstrated in the purification of fluorescently derivatized N-glycans (from human serum). Cobalt ferrite was found to be the most effective. Owing to the easy preparation and the simplicity of the magnetic separation the pure cobalt ferrite, magnetic nanoparticles could be efficient tools for the selective enrichment of serum N-glycans in HPLC measurements.

## 1. Introduction

Glycosylation is a post-translational modification of proteins, playing a key role in several biological processes [1,2]. The analysis of protein glycosylation is a multi-step procedure, in which fluorescent labeling is needed during sample preparation after the release of glycans from proteins [3]. Removal of fluorescent dye from glycan molecules is an essential step before the quantitative analysis of the glycans using high-performance liquid chromatography (HPLC) [4]. Several methods have been developed in recent years for glycan purification, such as solid-phase extraction, precipitation, paper chromatography and gel filtration [5]. However, the large elution volume and the necessity of pre-concentration is still a serious issue in these methods. Magnetic nanoparticles offer an easy way to avoid the pre-concentration process [6]. The most common magnetic nanomaterials in biological applications, such as magnetic resonance imaging (MRI), detoxification, drug delivery and hyperthermia, are the magnetic iron oxides, namely magnetite (Fe_3_O_4_) and maghemite (γ-Fe_2_O_3_). These nanoparticles can also be used in derivatized or non-modified form [7,8,9]. Other, different magnetic nanoparticles are available in addition to magnetite and maghemite, which are efficiently applicable in several biological and analytical processes (i.e., CrO_2_, MFe_2_O_4_). The substituted magnetic spinel ferrites MFe_2_O_4_ (M = Zn^2+^, Mn^2+^, Co^2+^, Ni^2+^, Mg^2+^, etc.) can be doped within a wide range with other metals, rare earth metals and other transition metals [10,11,12,13,14]. Thereby, their magnetic and structural properties may govern. The transitional metal containing ferrites, e.g., manganese ferrite (MnFe_2_O_4_) and cobalt ferrite (CoFe_2_O_4_), are successfully usable for improvements in hyperthermia applications, photothermal therapy and immunotherapy for cancer treatment, and are able to cause necrosis of murine breast cancer cells [15,16,17]. The interest in magnetic particles is also growing in the area of the glycan analytics; maltose-functionalized and glutathione-capped hydrophilic magnetic particles are efficient for glycopeptide enrichment [18,19]. There are several methods for the preparation of the magnetic ferrite nanoparticles, including hydrothermal [20,21], sonochemical [22,23], sol-gel [24,25], precipitation [26,27], micro-emulsion [28,29] and even mechanical alloying [30,31] methods. In our method, combustion and the sonochemical treatment were combined for the efficient synthesis of magnetic spinel nanoparticles. The essence of the sonochemical method is the exposure of the liquid medium to intense ultrasonic effects, and the induced sound waves create cycles of high and low pressure. Thus, the vapor pressure of the solvent decreases momentarily, which results in the formation of bubbles of a few micrometers in the mixture. These bubbles pulsate and grow until they reach a higher pressure range in the liquid, where they will collapse as the pressure increases [32]. At this point (“hot spot”), a huge amount of energy is released, causing the medium (in our case, it is polyethylene glycol) to act as a reducing agent in the reaction and initiate the formation of metal, metal oxide and metal hydroxide particles from the metal precursors. This method was supplemented by a combustion step to prepare magnetic NiFe_2_O_4_ and CoFe_2_O_4_ particles, which were compared in the glycan purification method. Commercial magnetic separation kits, for example, contain silicon-coated magnetic beads (Fe_3_O_4_@SiO_2_) [33]. Synthesis of these spherical, magnetic, composite particles is time-consuming, complicated, consists of many steps and even has a relatively low yield. In contrast, our aim was to develop a cheaper, faster, simpler process, which is also easy to scale-up for production. In this sense, our method contributes to an efficient and economical glycan enrichment process in HPLC analysis.

## 2. Results and Discussion

**Figure 1:**
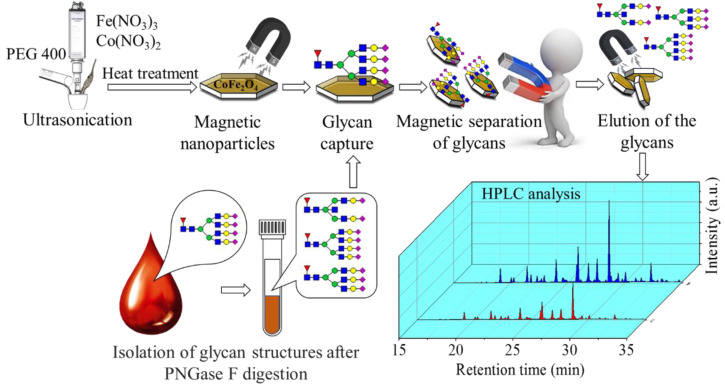
Scheme of the preparation of the ferrite nanoparticles, and their application in magnetic glycan separation method.

The aims and results of this study are summarized in Figure 1.

The synthesis of the magnetic nanoparticles involved a sonochemical activation step, in which highly dispersed metal hydroxide nanoparticles formed from the metal nitrate precursors in polyethylene-glycol-based dispersion. PEG400 was used in this study since it is a viscous liquid at room temperature and not a waxy solid, as with the higher-molecular-weight PEGs; moreover, it gave the desired small (metal oxide/hydroxide) particle size. The chemical effects of ultrasound irradiation are, primarily, attributed to its acoustic cavitation: the formation, growth and implosive collapse of bubbles in the irradiated liquid. During the collapse, there is a high energy density from the conversion of the kinetic energy of the liquid’s motion into the heating of the contents of the bubble, which can cover the energy requirements of chemical reactions, such as the formation of metal hydroxides from their nitrate precursors in the polyol phase. It is evident from the data in the Experimental section that iron(III) was added to Ni(II) or Co(II) in a 2:1 molar ratio to obtain the favored spinel structure with the following formula: MFe_2_O_4_ (where M stands for Mn, Cd, Co, Ni, Cu, Zn, etc.). Deviation from the stoichiometric form of the spinel structure may result in the formation of undesirable, non-magnetic metal oxides (i.e., CoO, Fe_2_O_3_ and NiO) in greater amounts.

In the second step, during the combustion method, the PEG-based colloid system of the iron and cobalt or nickel hydroxides was heated in a furnace in the presence of an air atmosphere at four different temperatures (573 K, 623 K, 673 K and 773 K). The duration of the heat treatment was 3 h, in each case. After burning of the PEG and the dehydration of the metal hydroxide nanoparticles, the expected spinel structures with magnetic properties formed.

**Figure 2:**
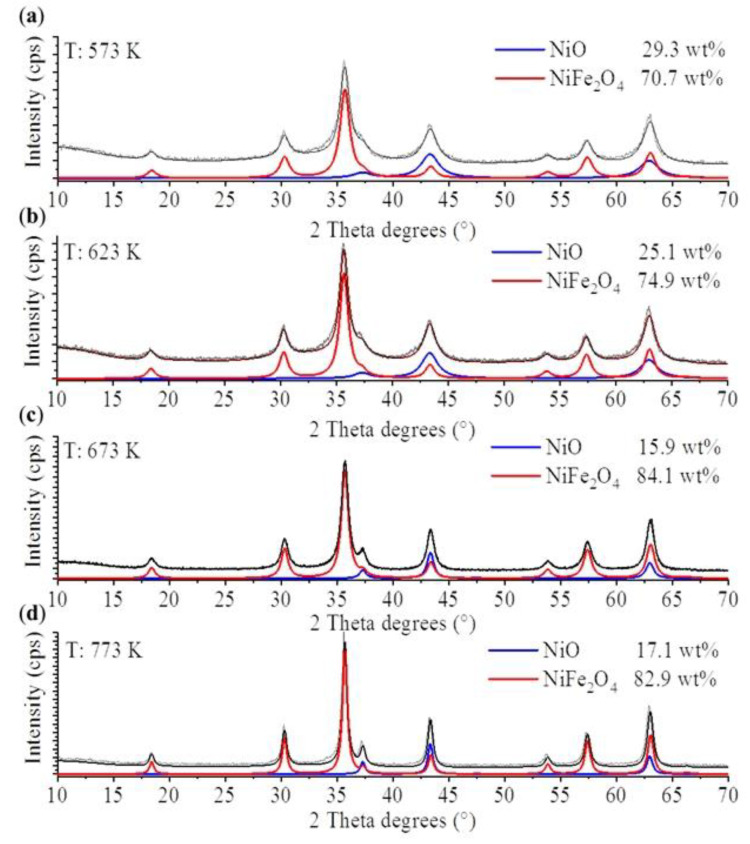
Rietveld refined X-ray diffractograms of the nickel ferrite (NiFe_2_O_4_) samples formed at different temperatures. (**a**) 573 K, (**b**) 632 K, (**c**) 673 K, (**d**) 773 K.

The identification and quantitative analysis of the different oxide phases in the magnetic spinel nanoparticles was carried out by X-ray diffraction (XRD) measurements. In the nickel-containing samples, besides the nickel ferrite, nickel(II) oxide was also identified (Figure 2). On the diffractograms, high-intensity peaks are found at 18.3° (111), 30.1° (220), 35.6° (311) 37.0 (222); 43.9° (400), 53.2° (422), 57.1° (511) and 63.7° (440) two Theta degrees, which correspond to NiFe_2_O_4_ (PDF: 54-0964). The amount of the spinel phase was relatively high in all samples, but the highest, 84.1 wt% was detected in the nanopowder formed at 673 K temperature. All samples contained NiO, which was confirmed based on the presence of peaks at 37.4° (111), 43.5° (200) and 63.1° (220) two Theta degrees, respectively (PDF 47-1049). The samples with the highest ferrite content were selected for the separation of glycans. It should be noted, however, that the nickel ferrite content of the samples prepared at 673 and 773 K was very similar: 84.1% and 82.9%, respectively. This may suggest that higher ferrite content may be obtained at temperatures between 673 and 773 K, if the ferrite content temperature curve has a maximum in this range. Otherwise, 673 K is the optimal temperature.

The mean particle size of the different oxide forms was calculated based on the XRD analysis. A positive correlation between the crystallite size and heat treatment temperature was found: higher temperatures yielded larger particles (Table 1). All samples were polydisperse, containing both ferrite and nickel oxide nanoparticles of similar size (Figure S1).

**Figure 3:**
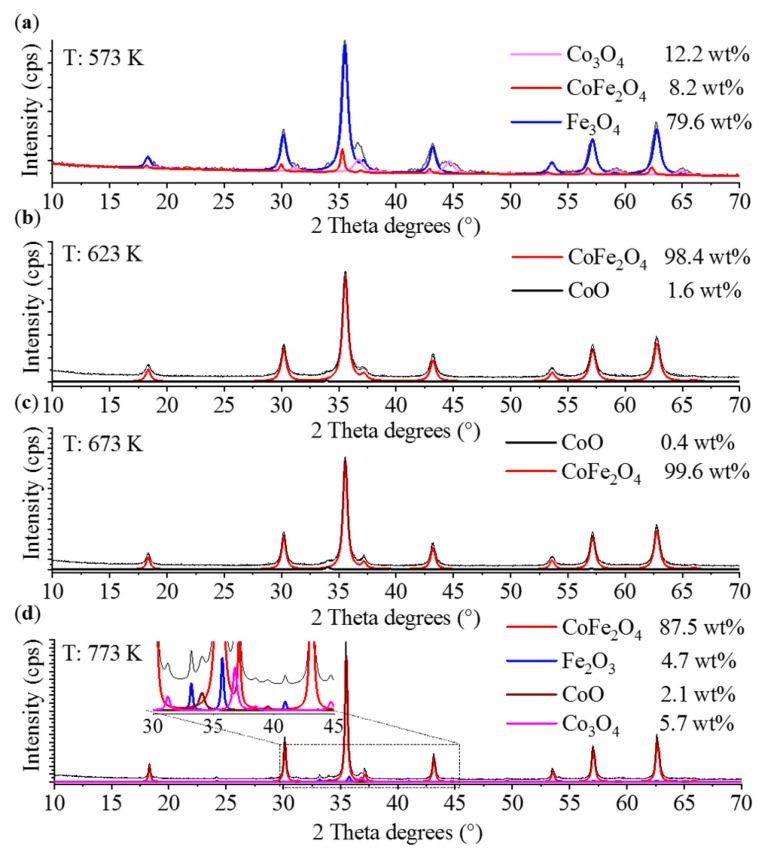
Rietveld refined X-ray diffractograms of the cobalt ferrite samples formed at different temperatures. (**a**) 573 K, (**b**) 632 K, (**c**) 673 K, (**d**) 773 K.

In the case of the cobalt ferrite, seven reflection peaks were identified at 18.2° (101), 30.2° (200), 35.8° (211), 43.0° (220), 54.1° (312), 57.3° (303) and 62.7° (224) two Theta degrees (PDF 22-1086), which supports the presence of cobalt ferrite in the samples prepared at four different temperatures (573 K, 623 K, 673 K and 773 K) (Figure 3). The highest amount of spinel phase (99.6 wt%) was detected in the cobalt ferrite prepared at 673 K. This almost pure cobalt ferrite, with negligible amounts of cobalt(II) oxide, is favorable in magnetic separation applications, since there is no material loss owing to the possible separation of the CoO particles. In the other samples, the presence of both cobalt(II) and cobalt(IV) oxide was detected. The reflections of the CoO phase were found at 33.9° (111), 39.4° (200) and 56.9° (220) two Theta degrees (PDF 42-1300). The quantity of this oxide was very low (0.4–2.1 wt%). However, the amount of the cobalt(IV) oxide was found to be higher (5.7–12.2 wt%) in the samples produced at 573 and 773 K, respectively. The reflections at 18.9° (111), 31.1° (220), 36.7° (311), 44.7° (400), 55.5° (422), 59.2° (511) and 65.0° (440) belonged to the Co_3_O_4_ phase (PDF 42-1467). Interestingly, magnetite was identified as the main phase (79.7 wt%) of the sample synthetized at 573 K. The characteristic reflections were visible at 18.3° (111), 30.2° (220), 35.5° (311), 43.32° (400), 53.6° (422), 57.1° (511) and 62.3° (440) two Theta degrees (PDF 19-629). One additional iron oxide phase, hematite, was also present in the sample produced at 773 K, justified by the appearance of the reflections at 24.2° (012), 33.8° (104), 35.7° (110), 40.9° (113), 49.6° (024), 54.1° (116), 62.6° (214) and 64.2° (300) corresponding to Fe_2_O_3_ (PDF 33-0664). Based on the XRD analysis, it could be established that the sample that was heat-treated at 673 K contained the magnetic phase (CoFe_2_O_4_) in the highest proportion, and the amount of CoO was negligible. Consequently, for further investigations, i.e., glycan separation assays, this sample was used.

The particle sizes were calculated based on the XRD measurements (Table 2). Similar to nickel ferrite, increasing temperature led to the formation of larger ferrite nanoparticles. It should be noted, however, that up to 673 K, the crystallite sizes were similar (around 16 and 18 nm), but the highest temperature (773 K) yielded much larger ferrite particles, which were more than double the mean particle size. The opposite trend was observed in the case of CoO: the particle size decreased with increasing temperature.

**Figure 4:**
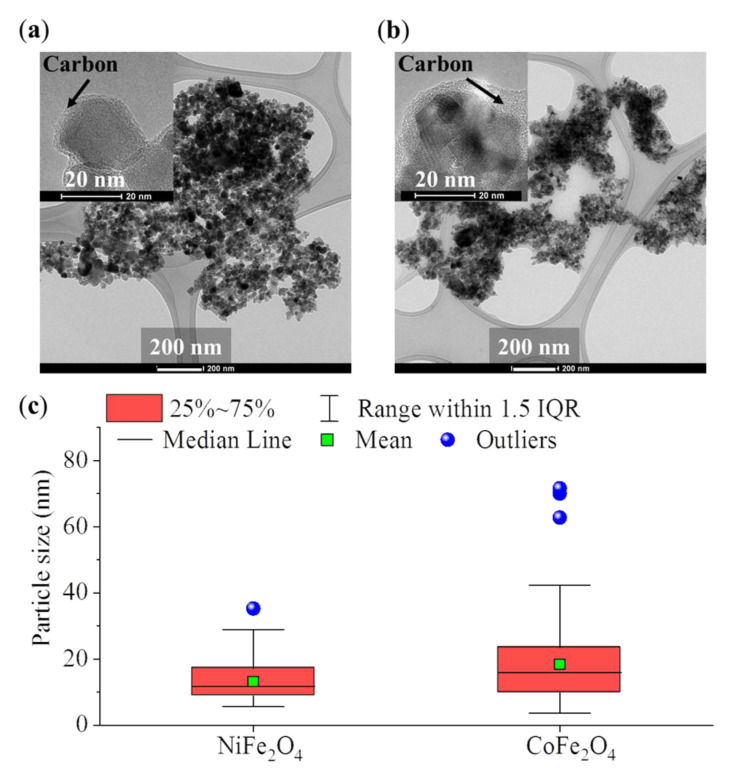
TEM images of the NiFe_2_O_4_ (**a**) and the CoFe_2_O_4_ (**b**) and the carbon layer on their surfaces. Box plot diagram of the particle size analysis (**c**).

The TEM images of nickel and cobalt ferrite (synthesized at 673 K) revealed the presence of small-sized ferrite nanoparticles (Figure 4a,b and Figure S1). The samples also contained such nanoparticles whose surfaces were embedded in an amorphous layer, namely the carbon from the burning of the PEG. The presence of this carbon layer was further supported by FT-IR measurements. To determine the exact carbon content of the samples formed at 673 K, CHNS element analysis was carried out. The carbon content was found to be very low in both cases: nickel ferrite contained 0.6 wt% carbon, while in the case of cobalt ferrite, 0.2 wt% carbon was found. Size distribution analysis was carried out using the pixel ratios of the particles and those of the scalebar on the TEM images (Figure 4c). As can be seen from the box plot diagram, the NiFe_2_O_4_ and the CoFe_2_O_4_ particle size distributions overlapped, and their mean values (the average diameters of the NiFe_2_O_4_ and the CoFe_2_O_4_ particles are also shown in Table 3 as 13.2 ± 6.3 nm and 18.4 ± 12.7, respectively) slightly differed from each other. To determine the significance of the 5.2 nm of mean difference, an ANOVA test was carried out and it was found that these mean values can be considered significantly different at a 95% confidence level. It is also worth mentioning that these size parameters were consistent with those derived from XRD measurements (10 ± 3 nm for NiFe_2_O_4_ shown in Table 1 and 18 ± 4 nm for CoFe_2_O_4_ shown in Table 2).

The detailed size statistics from the TEM results are more informative than the mean particle size, which was calculated based on Sherer’s method (Table 3) [34].

In the case of the nickel ferrite sample, the size distribution was tight and only two particle sizes were identified as outliers in the analysis; the average and median particle size were very close to each other. On the other hand, the cobalt ferrite size distribution was more inclined towards larger values, as shown by the increase in outliers present for this sample. This different behavior can be also demonstrated by the increasing differences when moving towards higher percentile values (from median to P95) in Table 3 for the two samples.

Surface polarity and the degree of dispersion in the liquid phase are key factors for efficient magnetic separation applications. The functional groups on the surface determine the tendency of the nanoparticles to form a stable dispersion. Therefore, it was necessary to investigate the oxygen-containing functional groups on the surface of the ferrite nanoparticles. Similar to the other cases, only the samples with the highest ferrite phases (produced at 673 K) were subjected to FT-IR measurements.

**Figure 5:**
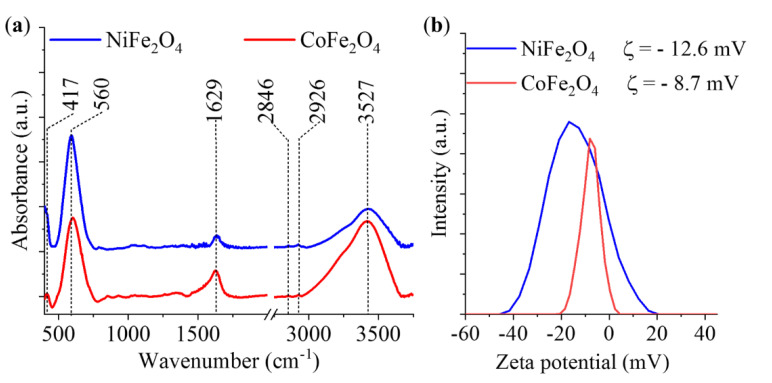
FT−IR spectrum (**a**) and the Zeta potential distribution (**b**) of the NiFe_2_O_4_ and CoFe_2_O_4_.

In the FT-IR spectra of the ferrite nanoparticles, two bands can be identified, which belong to the tetrahedral complexes (between 500 and 600 cm^−1^ wavenumbers), and to the octahedral complexes (between 400 and 450 cm^−1^) of the spinel structures (Figure 5a). The band at 560 cm^−1^ can be assigned to the vibration of the Fe^3+^–O^2−^ in the sublattice A site. On the other hand, the band at the lower wavenumber (417 cm^−1^) represents the trivalent metal–oxygen vibration at the octahedral B sites [35]. The spectra of both ferrite samples contained a band at 1629 cm^−1^, which can be assigned as surface hydroxyl group bending vibration. Two miniscule peaks are found at 2864 and 2926 cm^−1^, characteristic of the symmetric and asymmetric stretching vibration modes of the aliphatic and aromatic C–H bonds. These bands may be the result of the unburnt PEG derivatives on the particles’ surface. Finally, the wide absorption band in the region of 3000–3750 cm^−1^ belongs to the stretching vibration of hydroxyl groups (-OH, surface).

Zeta potential measurements were carried out in the case of the two ferrites (673 K) in distilled water (Figure 5b). The average Zeta potentials were −12.6 mV (NiFe_2_O_4_) and −8.7 mV (CoFe_2_O_4_); the negative surface charge can be attributed to the deprotonation of the hydroxyl groups. The role of hydroxyl groups is twofold. On the one hand, their presence improves the hydrophilic (polar) nature, resulting in better wettability, and enhances the electrostatic repulsion between the nanoparticles, which are key factors of good dispersibility. On the other hand, OH groups allow the formation of hydrogen bonds between the ferrite particles and glycan molecules, since the glycan molecules also contain hydroxyl functional groups. These hydrophilic (-OH) surfaces are assumed to be excellent adsorbents for the reversible (physical) binding of carbohydrate derivatives, from an aqueous mobile phase with high organic solvent content (acetonitrile) [36]. Thereby, the adsorbed glycans on the ferrite particles can be isolated from the supernatant by applying an external magnetic field. The release of the bound glycans from the surface of ferrite particles can be facilitated by an increase in the water content in the organic phase, by disrupting the hydrogen bonds between the adsorbate and the adsorbent. It should also be noted, however, that not only H-bonding but several other sorption mechanisms can play a role in the adsorption of carbohydrates on the surface of the ferrites, including ion exchange, surface complexation and cation bridging [37].

### Comparison of the Efficacy of the Nickel Ferrite and Cobalt Ferrite Magnetic Particles in Glycan Separation

The large surface area and strong but reversible adsorption capability, combined with their magnetic properties, make ferrites excellent materials in separation applications, such as the concentration of glycans from aqueous solutions. However, the non-magnetic phases, such as hematite, nickel oxide and cobalt oxides, can also bind the glycans, leading to low yield separations since the non-magnetic phases cannot be recollected by the magnetic field. Therefore, only the samples with the highest ferrite content were chosen for testing of glycan binding. These were NiFe_2_O_4_ and CoFe_2_O_4_ prepared at 673 K with 84.1% and 99.6% magnetic content, respectively.

**Figure 6:**
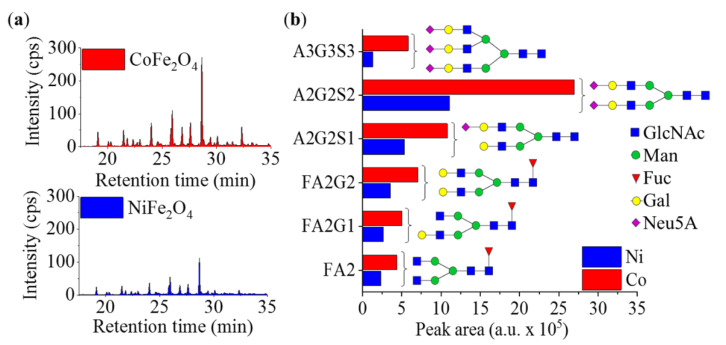
(**a**) Chromatograms of the glycan samples purified by cobalt ferrite and nickel ferrite; (**b**) Integrated peak areas of the chromatograms in case of the six most intensive glycan peaks.

The glycans were obtained from human serum, according to the PNGase F digestion protocol, and were fluorescently labeled by procainamide in the presence of picoline borane [9,38]. Before glycan separation, the dispersion and surface activation of the synthetized ferrite nanoparticles were carried out using HPLC water. The wetted and separated nanoparticles were suspended in the labeled glycan solution, the polarity of which was reduced by the addition of acetonitrile. After binding, the nanoparticles were removed using an external magnet, washed with 90% acetonitrile and eluted by HPLC water. After magnetic separation, the supernatant was analyzed by ultra-performance hydrophilic interaction liquid chromatography (UPLC) with online fluorescence detection. The resulting chromatograms, along with the comparison of the peak areas, are shown in Figure 6a,b; in addition, the names of the identified glycans can be found in Table 4.

Two main conclusions can be drawn from the data of Figure 6a,b. Firstly, a number of glycans could be well recovered from the serum (not all of them could be identified, however, since human serum contains a very complex mixture and not all the standards for identification were available); secondly, the glycan peaks recorded in the case of the two tested ferrite nanoparticles showed significant intensity differences. Based on both peak intensity and peak area data, the CoFe_2_O_4_ particles were found to be more efficient in the binding of the carbohydrates than the NiFe_2_O_4_.

## 3. Materials and Methods

### 3.1. Materials

For the synthesis of the magnetic ferrite-based nanoparticles, nickel(II) nitrate hexahydrate, Ni(NO_3_)_2_∙6H_2_O, MW: 290.8 g/mol (ThermoFisher GmbH, 76870 Kandel, Germany); cobalt(II) nitrate hexahydrate, Co(NO_3_)_2_∙6H_2_O, MW: 291.0 g/mol; and iron(III) nitrate nonahydrate, Fe(NO_3_)_3_∙9H_2_O, MW: 404.0 g/mol from the VWR Int. LtD. (B-3001 Leuven, Belgium) were used. Polyethylene glycol (PEG 400, Mw: ~400 Da, which corresponds to a chain length of approximately 8–9 links) from VWR Int. Ltd. (F-94126 Fontenay-sous-Bois, France) was used as the reducing agent and dispersion medium of the metal precursors. Acetonitrile, ammonium hydroxide, acetic acid, formic acid, picoline borane, procainamide and human serum were purchased from Sigma-Aldrich (St. Louis, MO, USA). PNGase F was purchased from New England Biolabs (Ipswich, MA, USA).

### 3.2. Methods

#### 3.2.1. Preparation of the Magnetic Spinel Nanoparticles

Cobalt- and nickel-containing ferrite nanoparticles were synthesized by using a two-step process, which was composed of sonochemical treatment followed by combustion. In the first step, iron(III) nitrate nonahydrate and one of the precursors (Table 5) were dissolved in 20 g polyethylene glycol and the solutions were treated by using a Hielscher UIP1000 Hdt high-intensity ultrasound homogenizer for 3 min (130 W, 19 kHz), equipped with a Bs4d22 ultrasonic block sonotrode (D: 22 mm).

The color of the dispersions deepened and changed to brownish red, indicating the formation of metal hydroxides from their corresponding nitrate salts.

In the second step, namely the combustion, the PEG-based colloid systems of the iron and cobalt or nickel hydroxides were heated in a furnace in the presence of an air atmosphere at four different temperatures (573 K, 623 K, 673 K and 773 K). The duration of the heat treatment was 3 h. After burning of the PEG and the dehydration of the metal hydroxide nanoparticles, the expected spinel structures with magnetic properties were formed.

#### 3.2.2. Characterization of the Magnetic Ferrite Nanoparticles

The size and morphology of the spinel nanoparticles were examined by high-resolution transmission electron microscopy (HRTEM, FEI Tecnai G2 electron microscope, 200 kV). The samples were prepared by dropping an aqueous suspension of the samples onto 300-mesh copper grids (Ted Pella Inc., Redding, CA, USA). X-ray diffraction (XRD) measurements using Powder Diffraction Files (PDFs) were used for the identification and the quantitative characterization of the different oxide phases. The diffractograms were processed by Rietveld refinement. A Bruker Discovery diffractometer (Cu-Kα source, 40 kV and 40 mA) in parallel beam geometry (Göbel mirror) with a Vantec detector was applied. The average crystallite size of the oxide domains was calculated by the mean column length calibrated method by using full width at half maximum (FWHM) and the width of the Lorentzian component of the fitted profiles. For the evaluation, TOPAS 4 software was used.

Zeta potentials of the magnetic ferrite nanoparticles were measured in aqueous dispersion based on their electrophoretic mobility (by laser Doppler electrophoresis). Malvern Zetasizer Nano ZS DLS equipment was applied.

The surface functional groups of the spinel particles were identified with Fourier transform infrared spectroscopy (FT-IR) by using a Bruker Vertex 70 spectroscope in transmission mode. During sample preparation, a 10 mg ferrite sample was homogenized with 250 mg spectroscopic-grade potassium bromide and was pelletized.

The carbon content of the ferrite samples was measured by a Vario Macro CHNS elemental analysis instrument, and phenanthrene was applied as a standard (C: 93.538%, H: 5.629%, N:0.179%, S: 0.453%), obtained from Carlo Erba Inc. The carrier gas was helium (99.9990%), while oxygen (99.995%) was used for oxidation, and the samples were loaded into tin foils.

#### 3.2.3. Glycan Sample Preparation

Glycan release was performed using 9 µL of human serum, according to the PNGase F digestion protocol of New England Biolabs (Ipswich, MA, USA). The released glycans were labeled by the addition of 10 µL 0.37 M procainamide and 300 mM picoline borane in 70/30% (*v/v*) of dimethyl sulfoxide/acetic acid, incubating for 4 h at 65 °C.

#### 3.2.4. Sample Clean-Up

Nickel ferrite and cobalt ferrite magnetic nanoparticles were tested for the selective capture and elution of procainamide-labeled glycans released from human serum. First, 20 mg of the synthetized ferrite nanoparticles was dissolved in 1 mL of HPLC water and 200 µL was transferred to an Eppendorf tube. The nanoparticle-containing tube was placed onto a magnetic stand to remove the supernatant. Following this, 170 µL of acetonitrile was added to the labeled glycan solution and suspended with the (supernatant removed) nanoparticles while removed from the magnet. After binding, the suspension was placed onto the magnetic stand and the supernatant was removed. This was followed by a washing step using 200 µL 90% acetonitrile suspended with the nanoparticles and removed from the magnet. Finally, the samples were eluted by 100 µL HPLC water from the magnetic nanoparticles and analyzed by ultra-performance hydrophilic interaction liquid chromatography with online fluorescence detection.

#### 3.2.5. LC-FLR Analysis

The prepared N-glycans were analyzed by ultra-performance liquid chromatography (UPLC) equipped with a fluorescence detector on a Waters Acquity UPLC instrument under the control of Empower 3 chromatography software (Waters, Milford, MA). Separations were performed by a Waters BEH (Ethylene Bridged Hybrid) glycan column, 100 × 2.1 mm i.d., 1.7 µm particles, using a linear gradient of 70–55% acetonitrile at 0.4 mL/min in 30 min, using 50 mM ammonium formate pH 4.4 as the mobile phase. Samples were prepared in 70% acetonitrile 30% water and 15 µL was injected in all runs. Samples were maintained at 15 °C prior to injection and the separation temperature was 60 °C. The fluorescence detection excitation/emission wavelengths were λ_ex_ = 308 nm and λ_em_ = 359 nm, respectively.

## 4. Conclusions

NiFe_2_O_4_ and CoFe_2_O_4_ magnetic nanoparticles were synthesized by a sonochemical method combined with combustion. During the sonochemical step, in the presence of polyethylene glycol, iron(III) nitrate, cobalt(II) nitrate and nickel(II) nitrate precursors were converted to the corresponding metal (oxi)hydroxides by the action of the high energy released during ultrasonic cavitation, in the polyol as the dispersion phase. During the second (combustion) step, these oxide/hydroxide dispersions were heated, resulting in the burn-off of the polyethylene glycol, while water was eliminated from the metal oxy/hydroxide nanoparticles and spinel structures (NiFe_2_O_4_ and CoFe_2_O_4_) formed. The temperature of the combustion was found to affect the ferrite content, and therefore needed to be optimized. XRD measurements revealed that, besides ferrites, simple metal oxides also formed, especially at lower temperatures. For both nickel and cobalt ferrite, the optimal combustion temperature was 673 K, at which the highest ferrite content was found, at 84.1 wt% NiFe_2_O_4_ and 99.6 wt% CoFe_2_O_4_, respectively. XRD and electron microscopy also revealed that a small amount of carbon phase (approximately 0.5 wt%) remained on the surface of the nanopowders. The size distribution was relatively broad, and the mean particle sizes were 13.2 ± 6.3 nm (nickel ferrite) and 18.4 ± 12.7 nm (cobalt ferrite). FT-IR and Zeta potential measurements indicated the presence of surface OH groups, which aided the dispersion of the particles in water, while it can promote the adsorption of compounds capable of H-bond formation. The practical applicability of the magnetic nanopowders with the highest ferrite content was compared in glycan separation from human serum. By controlling the polarity of the medium (with acetonitrile), good adsorption of the glycans was observed on the surfaces of the nanoparticles. The glycan-bound nanoparticles could be removed with a magnet, and then the glycans could be released by the addition of water. UPLC analysis showed that a number of glycans could be selectively and effectively captured. Based on the chromatographic peak areas, the cobalt ferrite nanoparticles were found to be the most efficient.

## Figures and Tables

**Table 1:** Mean particle sizes of the identified phases based on XRD results.

	NiFe_2_O_4_	NiO
573 K	7 ± 2 nm	4 ± 1 nm
623 K	8 ± 2 nm	5 ± 1 nm
673 K	10 ± 3 nm	11 ± 3 nm
773 K	15 ± 3 nm	16 ± 4 nm

**Table 2:** Mean particle sizes of the identified phases based on XRD results.

	CoFe_2_O_4_	Fe_3_O_4_	Co_3_O_4_	CoO	Fe_2_O_3_
573 K	16 ± 5 nm	13 ± 3 nm	9 ± 2 nm		
623 K	14 ± 3 nm			40 ± 9 nm	
673 K	18 ± 4 nm			20 ± 5 nm	
773 K	41 ± 7 nm		21 ± 5 nm	14 ± 4 nm	41 ± 9 nm

**Table 3:** Mean particle sizes (nm) of the identified phases based on TEM results.

d [nm]	Mean	SD	Min.	Max.	Q 1	Median	Q 3	P 90	P 95
NiFe_2_O_4_ (673 K)	13.2	6.3	5.6	35.3	8.8	11.8	17.1	20.9	26.2
CoFe_2_O_4_ (673 K)	18.4	12.7	3.6	71.6	9.8	15.9	23.4	27.8	42.1

**Table 4:** The separated glycan types.

FA2	Fucosylated-bi-antennary glycan
FA2G1	fucosylated-bi-antennary mono-galactosylated glycan
FA2G2	fucosylated-bi-antennary bi-galactosylated glycan
A2G2S1	bi-antennary bi-galactosylated mono-sialylated glycan
A2G2S2	bi-antennary bi-galactosylated di-sialylated glycan
A3G3S3	tri-antennary tri-galactosylated tri-sialylated glycan

**Table 5:** Weight (g) of the reactants used during the preparation of the magnetic spinel samples.

	Fe(NO_3_)_3_ ∙ 9H_2_O	Ni(NO_3_)_2_ ∙ 6H_2_O	Co(NO_3_)_2_ ∙ 6H_2_O
NiFe_2_O_4_	2.78 g (6.88 mmol)	1.00 g (3.44 mmol)	-
CoFe_2_O_4_	2.77 g (6.88 mmol)	-	1.00 g (3.44 mmol)

## Data Availability

Data is available upon request from the corresponding authors.

## Supplementary Materials

It is available online at https://www.mdpi.com/article/10.3390/ijms23095081/s1.
