# Supplementary material for: Sonochemical Combined Synthesis of Nickel Ferrite and Cobalt Ferrite Magnetic Nanoparticles and Their Application in Glycan Analysis
Source: Int J Mol Sci. 2022 May 3;23(9):5081. doi: 10.3390/ijms23095081 (PMC9103833; doi:10.3390/ijms23095081)
Supplement: Supplementary file 1 [file ijms-23-05081-s001.zip › ijms-1696951-supplementary.pdf]

Supplementary Information

Sonochemical Combined Synthesis of Nickel-Ferrite and Cobalt-Ferrite Magnetic Nanoparticles and Their Application in Glycan Analysis

Ágnes M. Ilosvai[1], Dalma Dojcsák[2], Csaba Váradi[2], Miklós Nagy[1*], Ferenc Kristály[3], Béla Fiser[1], Béla Viskolcz[1] and LászlóVanyorek[1*]

[1] Institute of Chemistry, University of Miskolc, Miskolc-Egyetemváros, 3515 Miskolc, Hungary ; agnes.ilosvai.maria@uni-miskolc.hu (Á .M.I); kemiklos@uni-miskolc.hu (M.N.); kemfiser@uni-miskolc.hu (B.F.); bela.viskolcz@uni-miskolc.hu (B.V.); kemvanyi@uni-miskolc.hu (L.V.)

[2] Advanced Materials and Intelligent Technologies Higher Education and Industrial Cooperation Centre, University of Miskolc, 3515 Miskolc, Hungary; dalma.dojcsak@uni-miskolc.hu (D.D.); kemcsv@uni-miskolc.hu (C.V.)

[3] Institute of Mineralogy and Geology, University of Miskolc, Miskolc-Egyetemváros, 3515 Miskolc, Hungary; askkf@uni-miskolc.hu

* Correspondence: kemiklos@uni-miskolc.hu and kemvanyi@uni-miskolc.hu

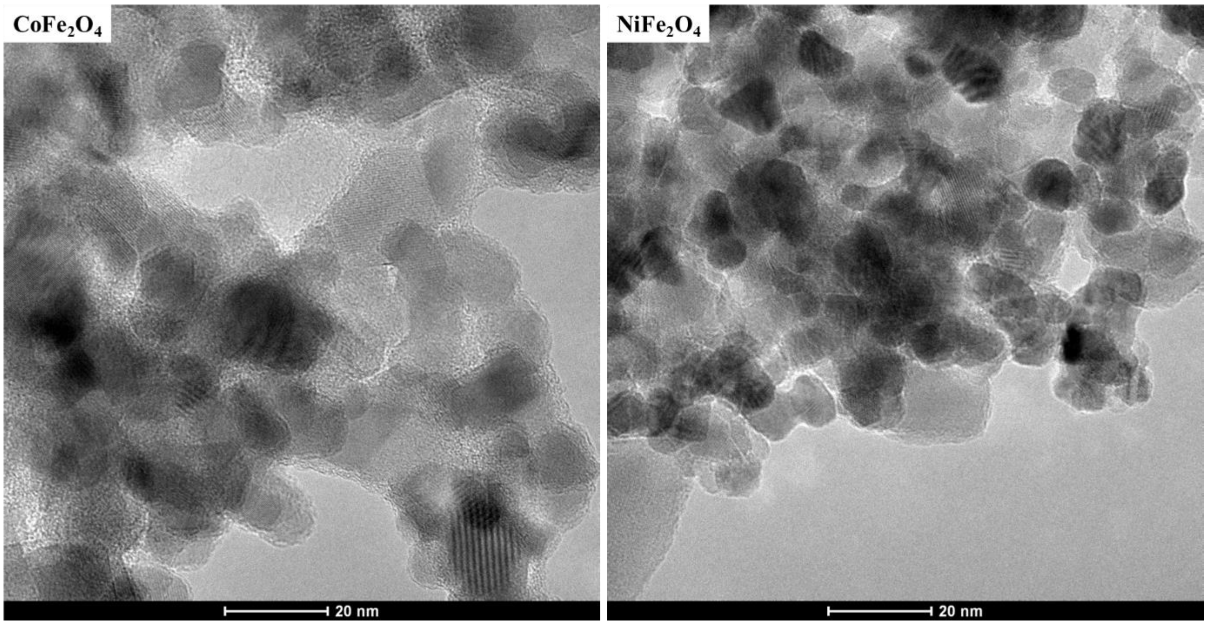

Figure S1. HRTEM pictures of the $NiFe_2O_4$ and $CoFe_2O_4$ nanoparticles.
